# The genome sequence of the killer whale, *Orcinus orca *(Linnaeus, 1758)

**DOI:** 10.12688/wellcomeopenres.18278.1

**Published:** 2022-10-07

**Authors:** Andrew Foote, Paulien Bunskoek, Wellcome Sanger Institute Tree of Life programme, Wellcome Sanger Institute Scientific Operations: DNA Pipelines collective, Tree of Life Core Informatics collective, Darwin Tree of Life Consortium

**Affiliations:** 1 Department of Natural History, NTNU University Museum, Norwegian University of Science and Technology (NTNU), Trondheim, Norway; 2 Dolfinarium Harderwijk, Harderwijk, Netherlands Antilles

**Keywords:** Orcinus orca, killer whale, genome sequence, chromosomal, Delphinidae

## Abstract

We present a genome assembly from an individual female *Orcinus orca *(the killer whale; Chordata; Mammalia; Artiodactyla; Delphinidae). The genome sequence is 2.65 gigabases in span. The majority of the assembly (93.76%) is scaffolded into 22 chromosomal pseudomolecules with the X sex chromosome assembled. The complete mitochondrial genome was also assembled and is 16.4 kilobases in length.

## Species taxonomy

Eukaryota; Metazoa; Chordata; Craniata; Vertebrata; Euteleostomi; Mammalia; Eutheria; Laurasiatheria; Artiodactyla; Whippomorpha; Cetacea; Odontoceti; Delphinidae; *Orcinus*; *Orcinus orca* (Linnaeus, 1758) (NCBI:txid9733).

## Background

The killer whale, *Orcinus orca*, is the largest and most widely geographically distributed species of dolphin (Delphinidae). Killer whales are found from the Arctic to the Antarctic, and all waters in between, occurring in the greatest densities at high latitudes ( Forney & Wade, 2007). In these areas of high biological productivity, they have evolved into ecotypes with different prey preferences. The best-studied of these being the fish-eating and mammal-eating ecotypes of the North Pacific ( Ford *et al.,* 1998). These two ecotypes differ not only in their diet, but also their behaviour and social structure ( Ford *et al.,* 1996). From the first study using restriction fragment length polymorphism (RFLP) ( Stevens *et al.,* 1989) or D-loop sequences ( Hoelzel & Dover, 1991) of mitochondrial DNA to complete mitochondrial genome sequences ( Morin *et al.,* 2010), and from microsatellites ( Barrett-Lennard, 2000; Hoelzel *et al.,* 1998) to whole genome sequences ( Foote *et al.,* 2016), the significant genetic differentiation between the two North Pacific ecotypes have been repeatedly and ever-more robustly established. The genetic impact of post-glacial colonisation of these waters by killer whales and the genetic signatures associated with divergence into distinct ecotypes have been a focus of genetic and genomic studies ( Foote *et al.,* 2016; Foote *et al.,* 2019; Foote *et al.,* 2021; Hoelzel *et al.,* 2007).

Genomic studies are also providing insights into the fitness and health of populations through the estimation of mutation load and inbreeding ( Foote *et al.,* 2021). Studies have found a small population of killer whales found in UK waters to show zero fecundity, consistent with high inbreeding ( Beck *et al.,* 2014), and genome sequencing revealed 38% of the genome comprising of runs of homozygosity in one female ( Foote *et al.,* 2021). The population now consists of just two adult males and is therefore beyond rescue ( Allendorf *et al.,* 2022). There are other populations of killer whales that seasonally occur in UK waters. These include groups that migrate each summer from Iceland to the Northeast of Scotland and Northern Isles of Orkney and Shetland, where they hunt seals close to shore ( Samarra & Foote, 2015). Genomic resources are key to monitoring the health, in terms of inbreeding, in these killer whale populations.

The existing draft genome assembly for the killer whale (Oorc_1.1) was generated as part of the first high-throughput sequencing project on marine mammal genomes ( Foote *et al.,* 2015). Prior to this, only a 2x coverage genome of a bottlenose dolphin, generated using the same methods as the first human genome project, was available as a reference ( Lindblad-Toh *et al.,* 2011). The Oorc_1.1 assembly came about following an unusual event. When a young female killer whale stranded in 2010 on the coast of the Netherlands, it provided the possibility to access fresh blood samples, collected by Dolfinarium Harderwijk for health checks. The priority was to extract DNA and sequence informative markers that could provide an indication of the population of origin of the killer whale, now named Morgan. This would be a key step, if releasing Morgan back to the wild was deemed possible. A combination of genetic analyses, and acoustic matching of Morgan’s vocal repertoire to recording databases (stereotyped call repertoires are culturally transmitted down matrilineal lineages in killer whale societies; Ford, 1991), pinpointed Morgan’s origins as the population that feeds upon the Norwegian spring-spawning herring, *Clupea harengus*, ( Vester & Samarra, 2011). The blood sample (BioSample: SAMN01180276) also provided sufficiently high molecular weight to generate the long-insert libraries needed for the first draft of the killer whale genome.

Whilst ultimately, the goal to rehabilitate and release Morgan back to the wild was unsuccessful, the genome assembly that resulted from that process has provided the backbone for several genomic studies which have contributed to our understanding of wild killer whale populations. The announcement by the Darwin Tree of Life project to generate high quality assemblies for all UK species provided an opportunity to improve the killer whale genome. Archived blood from Morgan once again provided the material, this time for generating Hi-C data and single molecule long-reads. The improved contiguity of this new DToL assembly will usher in a new phase of genomics research on killer whales, which will include identifying structural variants and phasing of population genomic data.

## Genome sequence report

**Figure 1.**
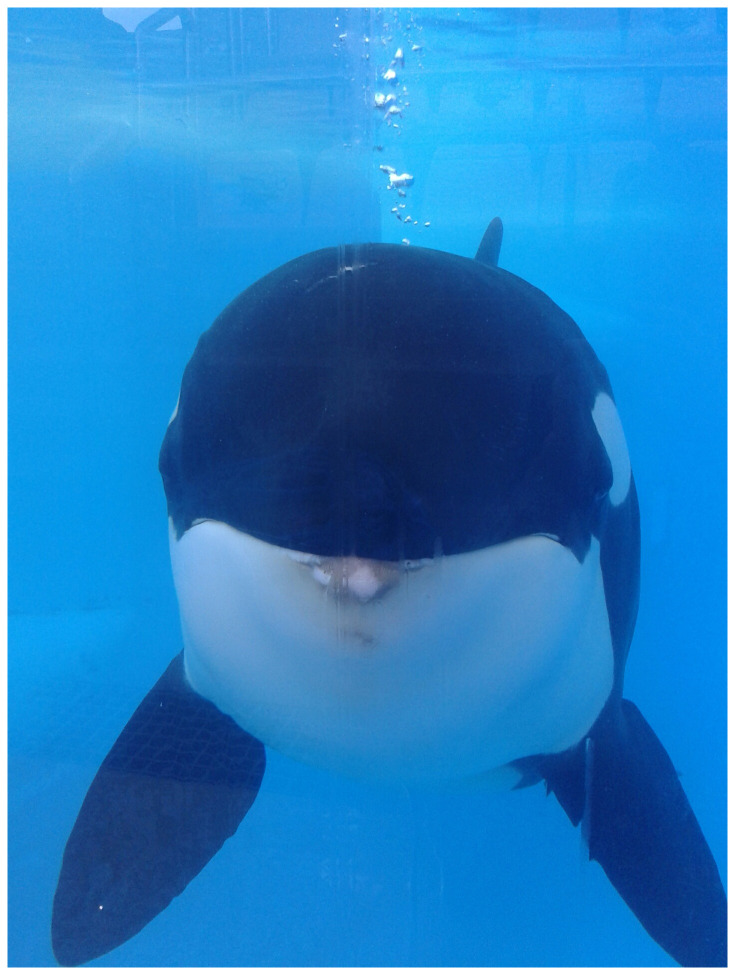
Image of Morgan, the *Orcinus orca* individual whose blood sample was used for the genome assembly. Morgan now resides at Loro Parque, Tenerife. Image credit: Dolfinarium, Harderwijk, Netherlands.

The genome was sequenced from a juvenile female *O. orca* collected from Dolfinarium, Harderwijk, Netherlands ( Figure 1). A total of 34-fold coverage in Pacific Biosciences single-molecule HiFi long reads were generated. Primary assembly contigs were scaffolded with chromosome conformation Hi-C data, used with permission from the DNA Zoo Consortium ( dnazoo.org). Manual assembly curation corrected 100 missing/misjoins, reducing the scaffold number by 13.87%, and increasing the scaffold N50 by 8.19%.

**Figure 2.**
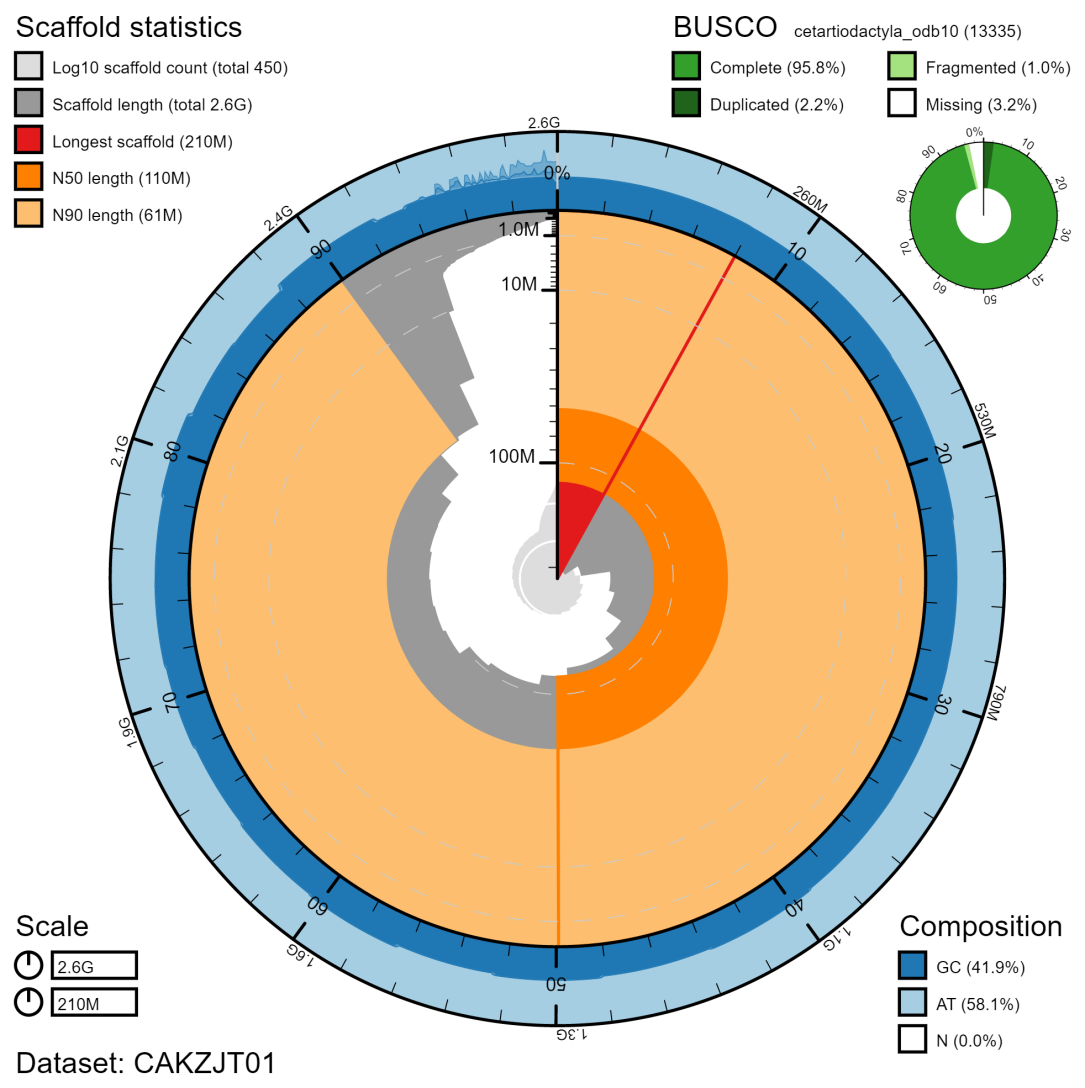
Genome assembly of *Orcinus orca*, mOrcOrc1.1: metrics. The BlobToolKit Snailplot shows N50 metrics and BUSCO gene completeness. The main plot is divided into 1,000 size-ordered bins around the circumference with each bin representing 0.1% of the 2,647,351,467 bp assembly. The distribution of chromosome lengths is shown in dark grey with the plot radius scaled to the longest chromosome present in the assembly (212,697,510 bp, shown in red). Orange and pale-orange arcs show the N50 and N90 chromosome lengths (114,219,206 and 61,273,037 bp), respectively. The pale grey spiral shows the cumulative chromosome count on a log scale with white scale lines showing successive orders of magnitude. The blue and pale-blue area around the outside of the plot shows the distribution of GC, AT and N percentages in the same bins as the inner plot. A summary of complete, fragmented, duplicated and missing BUSCO genes in the cetartiodactyla_odb10 set is shown in the top right. An interactive version of this figure is available at https://blobtoolkit.genomehubs.org/view/mOrcOrc1.1/dataset/CAKZJT01/snail.

The final assembly has a total length of 2.65 Gb in 447 sequence scaffolds with a scaffold N50 of 114.2 Mb ( Table 1). The majority, 93.76%, of the assembly sequence was assigned to 22 chromosomal-level scaffolds, representing 21 autosomes (numbered by sequence length) and the X sex chromosome ( Figure 2– Figure 5; Table 2). The order and orientation of scaffolds in a repetitive region of chromosome 17 (~33.6Mb) is uncertain.

**Table 1.** Genome data for *Orcinus orca*, mOrcOrc1.1.

*Project accession data*	
Assembly identifier	mOrcOrc1.1
Species	*Orcinus orca*
Specimen	mOrcOrc1 (genome assembly)
NCBI taxonomy ID	9733
BioProject	PRJEB51334
BioSample ID	SAMEA8800229
Isolate information	Blood sample (mOrcOrc1)
*Raw data accessions*	
PacificBiosciences SEQUEL II	ERR9127947-ERR9127951
*Genome assembly*	
Assembly accession	GCA_937001465.1
*Accession of alternate haplotype*	GCA_937001455.1
Span (Gb)	2.65
Number of contigs	570
Contig N50 length (Gb)	2.65
Number of scaffolds	447
Scaffold N50 length (Mb)	114.2
Longest scaffold (Mb)	212.7
BUSCO * genome score	C:95.8%[S:93.5%,D:2.2%],F:1.0%,M:3.2%,n:13,335

*BUSCO scores based on the cetartiodactyla_odb10 BUSCO set using v5.3.2. C= complete [S= single copy, D=duplicated], F=fragmented, M=missing, n=number of orthologues in comparison. A full set of BUSCO scores is available at https://blobtoolkit.genomehubs.org/view/mOrcOrc1.1/dataset/CAKZJT01/busco.

**Figure 3.**
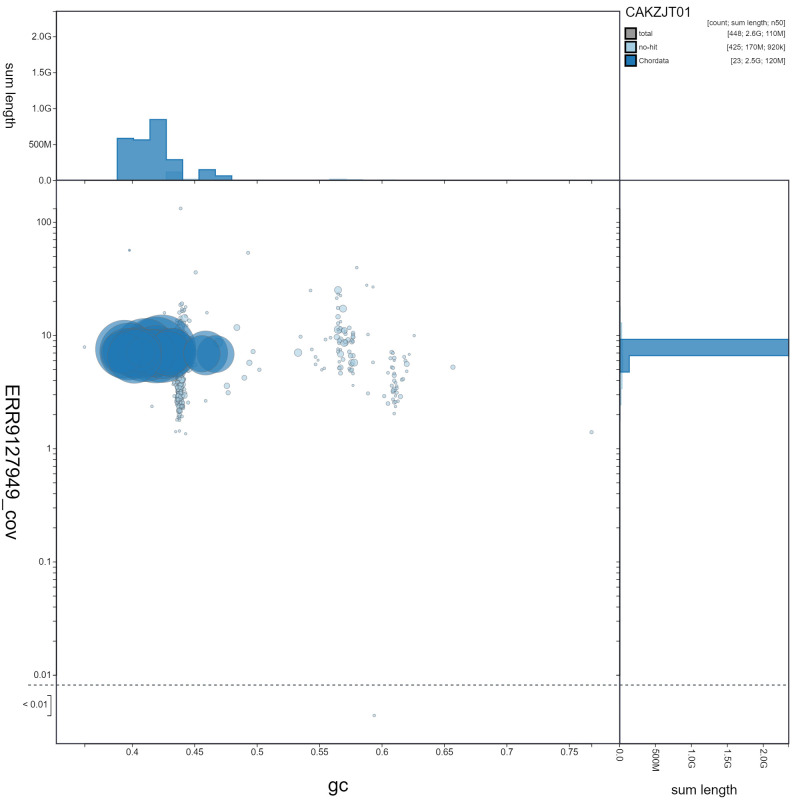
Genome assembly of *Orcinus orca*, mOrcOrc1.1: GC coverage. BlobToolKit GC-coverage plot. Scaffolds are coloured by phylum. Circles are sized in proportion to scaffold length. Histograms show the distribution of scaffold length sum along each axis. An interactive version of this figure is available at https://blobtoolkit.genomehubs.org/view/mOrcOrc1.1/dataset/CAKZJT01/blob.

**Figure 4.**
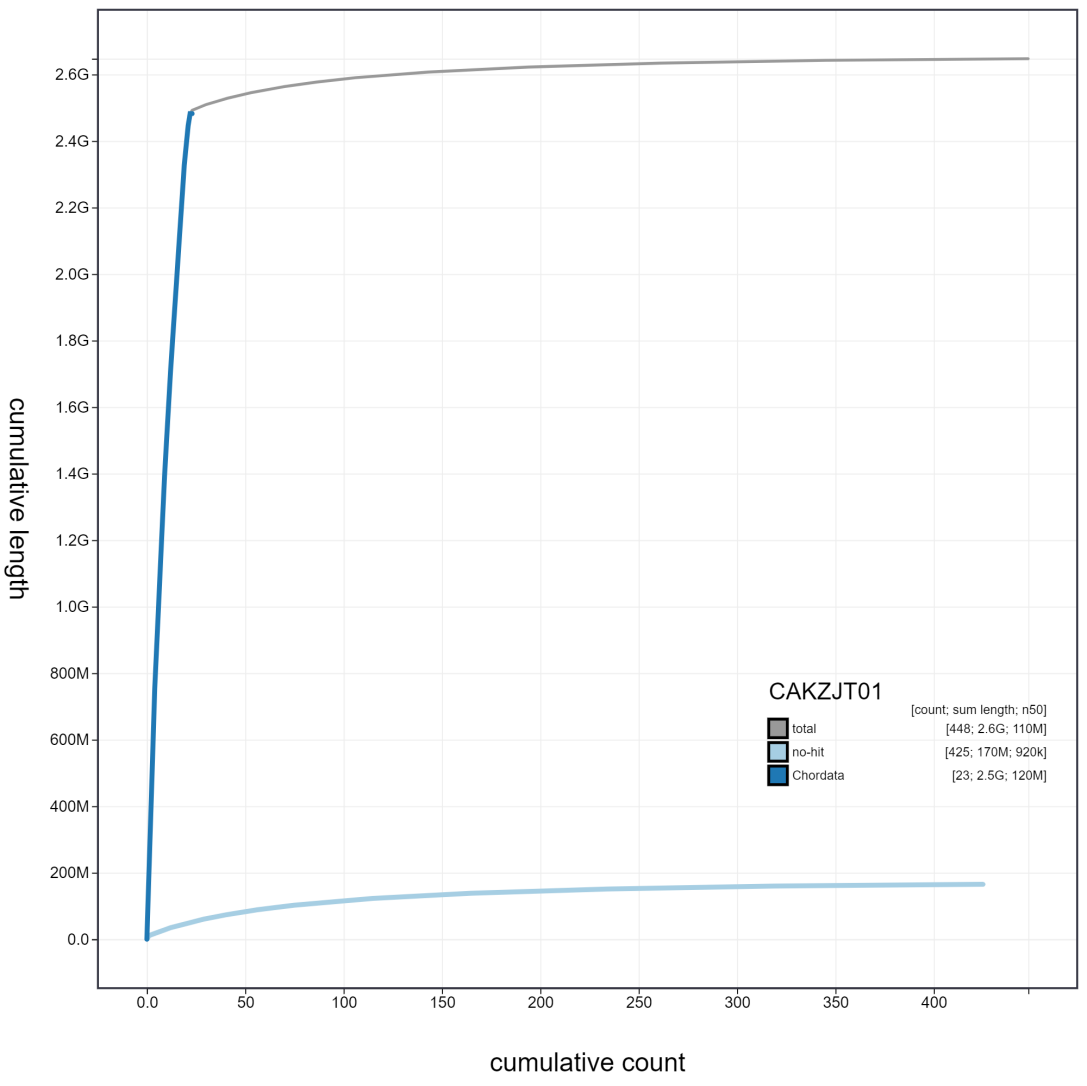
Genome assembly of *Orcinus orca*, mOrcOrc1.1: cumulative sequence. BlobToolKit cumulative sequence plot. The grey line shows cumulative length for all scaffolds. Coloured lines show cumulative lengths of scaffolds assigned to each phylum using the buscogenes taxrule. An interactive version of this figure is available at https://blobtoolkit.genomehubs.org/view/mOrcOrc1.1/dataset/CAKZJT01/cumulative.

**Figure 5.**
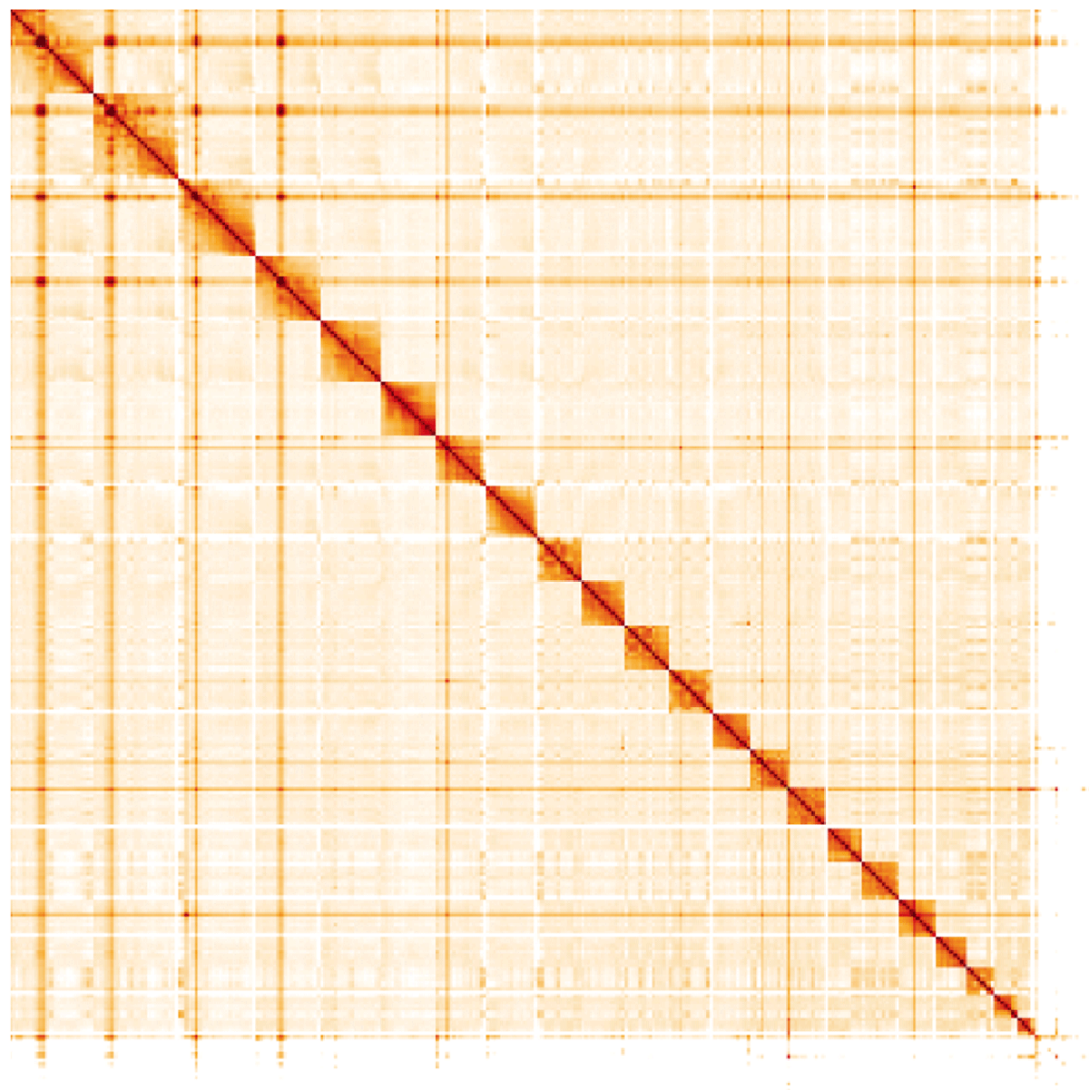
Genome assembly of *Orcinus orca*, mOrcOrc1.1: Hi-C contact map. Hi-C contact map of the mOrcOrc1.1 assembly, visualised in HiGlass. Chromosomes are arranged in size order from left to right and top to bottom. The interactive Hi-C map can be viewed at https://genome-note-higlass.tol.sanger.ac.uk/l/?d=VM4XqbhDQt6aK2-AY8Gsig.

**Table 2.** Chromosomal pseudomolecules in the genome assembly of *Orcinus orca*, mOrcOrc1.1.

INSDC accession	Chromosome	Size (Mb)	GC%
OW443360.1	1	212.7	42.4
OW443361.1	2	201.9	41.9
OW443362.1	3	185.2	41.0
OW443363.1	4	154.9	39.4
OW443364.1	5	149.3	39.7
OW443366.1	6	120.9	41.9
OW443367.1	7	120.8	40.4
OW443368.1	8	114.2	42.5
OW443369.1	9	105.9	40.0
OW443370.1	10	105.9	43.2
OW443371.1	11	102.9	41.6
OW443372.1	12	93.4	39.5
OW443373.1	13	91.5	41.6
OW443374.1	14	90.5	43.2
OW443375.1	15	89.6	42.9
OW443376.1	16	87.9	45.9
OW443377.1	17	87.5	40.6
OW443378.1	18	78.6	39.2
OW443379.1	19	61.3	46.7
OW443380.1	20	59.1	45.6
OW443381.1	21	35.4	40.7
OW443365.1	X	132.5	40.2
OW443382.1	MT	0.02	39.7
-	Unplaced	165.1	46.5

The assembly has a BUSCO v5.3.2 ( Manni *et al.,* 2021) completeness of 95.8% (single 93.5%, duplicated 2.2%) using the cetartiodactyla_odb10 reference set (n=13,335). While not fully phased, the assembly deposited is of one haplotype. Contigs corresponding to the second haplotype have also been deposited.

## Methods

### Sample acquisition and nucleic acid extraction

A juvenile female *O. orca*, later named Morgan, was found stranded on the Netherlands coast and identified by Andrew Foote (Norwegian University of Science and Technology). Morgan was transported to Dolfinarium, Harderwijk, Netherlands (latitude 52.3541, longitude 5.6172). A blood sample was drawn by a veterinarian and stored in a Paxgene blood vial before transportation on dry ice to Copenhagen where it was frozen at -80 degrees. DNA was extracted from the blood sample of mOrcOrc1 by Andrew Foote at the University of Copenhagen.

The pre-extracted DNA arrived at the Tree of Life laboratory, Wellcome Sanger Institute, courtesy of Andrew Foote. Fragment size analysis of 0.01–0.5 ng of DNA was then performed using an Agilent FemtoPulse. High molecular weight (HMW) DNA was extracted using the Qiagen MagAttract HMW DNA extraction kit. HMW DNA was sheared into an average fragment size between 12–20 kb in a Megaruptor 3 system with speed setting 30. Sheared DNA was purified by solid-phase reversible immobilisation using AMPure PB beads with a 1.8X ratio of beads to sample to remove the shorter fragments and concentrate the DNA sample. The concentration of the sheared and purified DNA was assessed using a Nanodrop spectrophotometer and Qubit Fluorometer and Qubit dsDNA High Sensitivity Assay kit. Fragment size distribution was evaluated by running the sample on the FemtoPulse system.

Hi-C data were generated at the Baylor College of Medicine from the blood sample of an unnamed female *O. orca* individual (BioSample: SAMN10973740; Dudchenko *et al.,* 2017) and used with permission from the DNA Zoo Consortium ( dnazoo.org).

### Sequencing

Pacific Biosciences HiFi circular consensus sequencing libraries were constructed according to the manufacturers’ instructions. Sequencing was performed by the Scientific Operations core at the Wellcome Sanger Institute on a Pacific Biosciences SEQUEL II (HiFi) instrument.

### Genome assembly

Assembly was carried out with Hifiasm ( Cheng *et al.,* 2021); haplotypic duplication was identified and removed with purge_dups ( Guan *et al.,* 2020). The assembly was then scaffolded with Hi-C data ( Rao *et al.,* 2014) using YaHS ( Zhou *et al.,* 2022). The assembly was checked for contamination as described previously ( Howe *et al.,* 2021). Manual curation was performed using HiGlass ( Kerpedjiev *et al.,* 2018) and Pretext. The mitochondrial genome was assembled using MitoHiFi ( Uliano-Silva *et al.,* 2021), which performs annotation using MitoFinder ( Allio *et al.,* 2020). The genome was analysed and BUSCO scores generated within the BlobToolKit environment ( Challis *et al.,* 2020). Table 3 contains a list of all software tool versions used, where appropriate.

**Table 3.** Software tools used.

Software tool	Version	Source
Hifiasm	0.16.1-r375	Cheng *et al.,* 2021
purge_dups	1.2.3	Guan *et al.,* 2020
MitoHiFi	2.0	Uliano-Silva *et al.,* 2021
YaHS	1.1.91eebc2	Zhou *et al.,* 2022
HiGlass	1.11.6	Kerpedjiev *et al.,* 2018
PretextView	0.2.x	https://github.com/wtsi-hpag/ PretextView
BlobToolKit	3.2.7	Challis *et al.,* 2020

## Data Availability

European Nucleotide Archive: Orcinus orca (killer whale). Accession number PRJEB51334; https://identifiers.org/ena.embl/PRJEB51334. The genome sequence is released openly for reuse. The *O. orca* genome sequencing initiative is part of the Darwin Tree of Life (DToL) project. All raw sequence data and the assembly have been deposited in INSDC databases. The genome will be annotated and presented through the Ensembl pipeline at the European Bioinformatics Institute. Raw data and assembly accession identifiers are reported in Table 1.

## Author information

Members of the Wellcome Sanger Institute Tree of Life programme are listed here: https://doi.org/10.5281/zenodo.6866293.

Members of Wellcome Sanger Institute Scientific Operations: DNA Pipelines collective are listed here: https://doi.org/10.5281/zenodo.5746904.

Members of the Tree of Life Core Informatics collective are listed here: https://doi.org/10.5281/zenodo.6125046.

Members of the Darwin Tree of Life Consortium are listed here: https://doi.org/10.5281/zenodo.6418363.
