# Supplementary material for: The genome sequence of the killer whale, Orcinus orca (Linnaeus, 1758)
Source: Wellcome Open Res. 2022 Oct 7;7:250. [Version 1] doi: 10.12688/wellcomeopenres.18278.1 (PMC10039322; doi:10.12688/wellcomeopenres.18278.1)
Supplement: Peer review history

# Peer review: Reviewer response for version 1

**DOI:** 10.21956/wellcomeopenres.20260.r54010

**Published:** 2023-03-24

**Authors:** Galaxia Cortés-Hinojosa

**Affiliations:** 1 Pontificia Universidad Católica de Chile, Santiago, Chile

The article called "The genome sequence of the killer whale, Orcinus orca (Linnaeus, 1758)" provides a nearly full genome sequence using a novel approach through HiFi long reads of Morga's genome, an *Orcinus orca* stranded in 2010. Morgan original genomic sequence is the base of relevant research in the fields of genomic and molecular ecology for this specie. The current paper is valuable research since improves the original data set and consequently will favor more investigations on the field.

Are sufficient details of methods and materials provided to allow replication by others?

Yes

Is the rationale for creating the dataset(s) clearly described?

Yes

Are the datasets clearly presented in a useable and accessible format?

Yes

Are the protocols appropriate and is the work technically sound?

Yes

Reviewer Expertise:

Virus and pathogen discovery, molecular virology, marine mammals ecology and conservation and veterinary sciences

I confirm that I have read this submission and believe that I have an appropriate level of expertise to confirm that it is of an acceptable scientific standard.

# Peer review: Reviewer response for version 1

**DOI:** 10.21956/wellcomeopenres.20260.r55424

**Published:** 2023-03-24

**Authors:** Michael Westbury

**Affiliations:** 1 University of Copenhagen, Copenhagen, Denmark

This manuscript presents a nice overview of previous work on the genetics of killer whales. I enjoyed the story about Morgan and her DNA and think it is great that her DNA can still be used to make such a high quality assembly, even after all these years. The only real comment I have is that it would be nice to have a bit more information on the parameters used during the assembly process as currently only the software names are mentioned.

Are sufficient details of methods and materials provided to allow replication by others?

Partly

Is the rationale for creating the dataset(s) clearly described?

Yes

Are the datasets clearly presented in a useable and accessible format?

Yes

Are the protocols appropriate and is the work technically sound?

Yes

Reviewer Expertise:

Genomics, evolution

I confirm that I have read this submission and believe that I have an appropriate level of expertise to confirm that it is of an acceptable scientific standard.

# Peer review: Reviewer response for version 1

**DOI:** 10.21956/wellcomeopenres.20260.r52724

**Published:** 2022-10-25

**Authors:** Jayan Senevirathna

**Affiliations:** 1 Department of Animal Science, Faculty of Animal Science and Export Agriculture, Uva Wellassa University, Badulla, Sri Lanka; 2 Laboratory of Aquatic Molecular Biology and Biotechnology, Department of Aquatic Bioscience, Graduate School of Agricultural and Life Sciences, The University of Tokyo, Tokyo, Japan

The work is exciting and genome data of female killer whales will be necessary. However, the following are some comments to be concerned about:

1. MT genome analysis can be included as a phylogeny. If the MT genome shows polymorphism, it can be significant.
2. Please show PacBio raw data information and reasons for combining Hi-C data. Table 1 should include a number of raw reads from PacBio and Hi-C incorporated data. Is the N50 of contigs equal to span/genome size...? Please check the BUSCO results of the contigs assembly.

Are sufficient details of methods and materials provided to allow replication by others?

Yes

Is the rationale for creating the dataset(s) clearly described?

Yes

Are the datasets clearly presented in a useable and accessible format?

Yes

Are the protocols appropriate and is the work technically sound?

Yes

Reviewer Expertise:

Aquatic molecular biology and biotechnology, Marine mammalogy, Aquatic biology and ecology, Zoology

I confirm that I have read this submission and believe that I have an appropriate level of expertise to confirm that it is of an acceptable scientific standard.
